# Awareness, behavior and attitudes concerning sun exposure among beachgoers in the northern coast of Peru

**DOI:** 10.7717/peerj.6189

**Published:** 2019-01-15

**Authors:** Carlos J. Toro-Huamanchumo, Sara J. Burgos-Muñoz, Luz M. Vargas-Tineo, Jhosuny Perez-Fernandez, Otto W. Vargas-Tineo, Ruth M. Burgos-Muñoz, Javier A. Zentner-Guevara, Carlos Bada

**Affiliations:** 1 Universidad de San Martín de Porres, Facultad de Medicina, Centro de Investigación en Epidemiología Clínica y Medicina Basada en Evidencias, Lima, Peru; 2 School of Medicine, Universidad de San Martín de Porres, Chiclayo, Peru; 3 School of Medicine, Universidad de San Martín de Porres, Lima, Peru; 4 Clínica San Judas Tadeo, Lima, Peru

**Keywords:** Health knowledge attitudes practices, Sunlight, Skin neoplasms, Ultraviolet rays

## Abstract

**Background:**

Skin cancer incidence has increased over the last years, becoming a major public health problem.

**Objective:**

To describe the awareness, behavior and attitudes concerning sun exposure among beachgoers in the northern coast of Peru.

**Methods:**

We conducted a cross-sectional study in the Pimentel beach, Peru. The “Beach Questionnaire” was used and we surveyed all the beachgoers from 8 a.m. to 4 p.m. and from March 5 to March 19. For the statistical analysis, sun exposure habits, sunburns history, knowledge, attitudes and practices were crossed with sex using the chi2 test.

**Results:**

We surveyed 410 beachgoers, the most frequent phototype was type III (40.5%). Only the 13.66% of the respondents correctly answered the seven knowledge questions related to sun exposure and skin cancer. Men more frequently agreed that “when they are tanned their clothes looks nicer” (*p* = 0.048). Likewise, regarding the questions “Sunbathing is relaxing” and “Sunbathing improves my mood”, men agreed or totally agreed with more frequency than women (63.64% vs. 46.15%, *p* < 0.001; and 61.36% vs 49.15%, *p* = 0.014, respectively). Regarding sun protection practices, women more frequently used sunshade (*p* = 0.001) and sunscreen (SPF ≥ 15) (*p* < 0.001) when compared to the male group.

**Conclusion:**

Sun exposure is a potentially preventable risk factor for skin cancer. Thus, awareness of the risks of UVR overexposure and adequate sun-protective behaviors and attitudes are essential. Our results, however, are not as favorable as expected. Public health efforts should encourage sun-safety precautions and intervention campaigns should be carried out in recreational settings, such as the beaches.

## Background

Skin cancer incidence has increased over the last years, becoming a major public health problem with a serious economic burden to the healthcare system of many countries (Erdmann et al., 2013; Garbe & Leiter, 2009; Guy et al., 2015). According to GLOBOCAN estimates, about 232.000 cases of melanoma and 55.000 deaths from this cause occurred worldwide in 2012 (Ferlay et al., 2015).

In recent years, global incidence rates of skin cancer have increased and there are some published reports that evidence this situation. For example, melanoma raw incidence rates per 100,000 US population has climbed from 22.2 to 23.6 (2009–2016 period). Similarly, raw mortality rates per 100 000 population has increased from 2.8 to 3.1 (Glazer et al., 2017). In Europe, melanoma trends has also increased in recent years, with the highest incidence rates in the UK, Ireland and the Netherlands (Arnold et al., 2014). Unfortunately, available data for Latin America is very limited (Schmerling et al., 2011). In Peru, there has been reported a growing trend of skin cancer, becoming the fourth most frequent type of cancer in the country (Sordo & Gutiérrez, 2013).

Sun exposure is considered a potentially preventable risk factor for skin cancer (Molho-Pessach & Lotem, 2007) and an adequate knowledge and good practices play an important role in the prevention of the disease. In fact, some studies have been carried out in order to assess these variables in patients, workers and students (Thomas-Gavelan et al., 2011; Lucena et al., 2014; Hault et al., 2016; Gao, Liu & Liu, 2014; Fernández-Morano et al., 2017; Fernández-Morano et al., 2014). However, only a few have focused on beachgoers, who are an important population at risk (Cercato et al., 2015; Pagoto, McChargue & Fuqua, 2003; Weinstock et al., 2000), and two of these studies only focused on behaviors and did not address knowledge or attitudes. In addition, the countries where these studies were conducted have a UV index lower than that reported in Peru (Newman & McKenzie, 2011).

In Peru, high temperature peaks have been reported over the last years, especially in 2017 (Servicio Nacional de Meteorología e Hidrología, 2017). In addition, Peru has been cataloged by the National Meteorology and Hydrology Service (SENAMHI) as one of the countries with the highest solar radiation, reaching an index of ultraviolet radiation (UV index) of 19 on a scale of 0 to 20 (Servicio Nacional de Meteorología e Hidrología, 2017). The northern coast of Peru has a semi-warm and tropical-dry climate where rainfall is barely present (Oficina Nacional de Gobierno Electrónico e Informática, 2017; Feddema, 2005). In summer, this region becomes even warmer, surpassing 30 °C (Servicio Nacional de Meteorología e Hidrología, 2017; Oficina Nacional de Gobierno Electrónico e Informática, 2017).

For the above mentioned, the objective of the present study was to describe the awareness, behavior and attitudes concerning sun exposure among beachgoers in the northern coast of Peru.

## Methods

### Study design

We conducted a cross-sectional study in the Pimentel beach, Peru.

### Setting and participants

**Figure 1:**
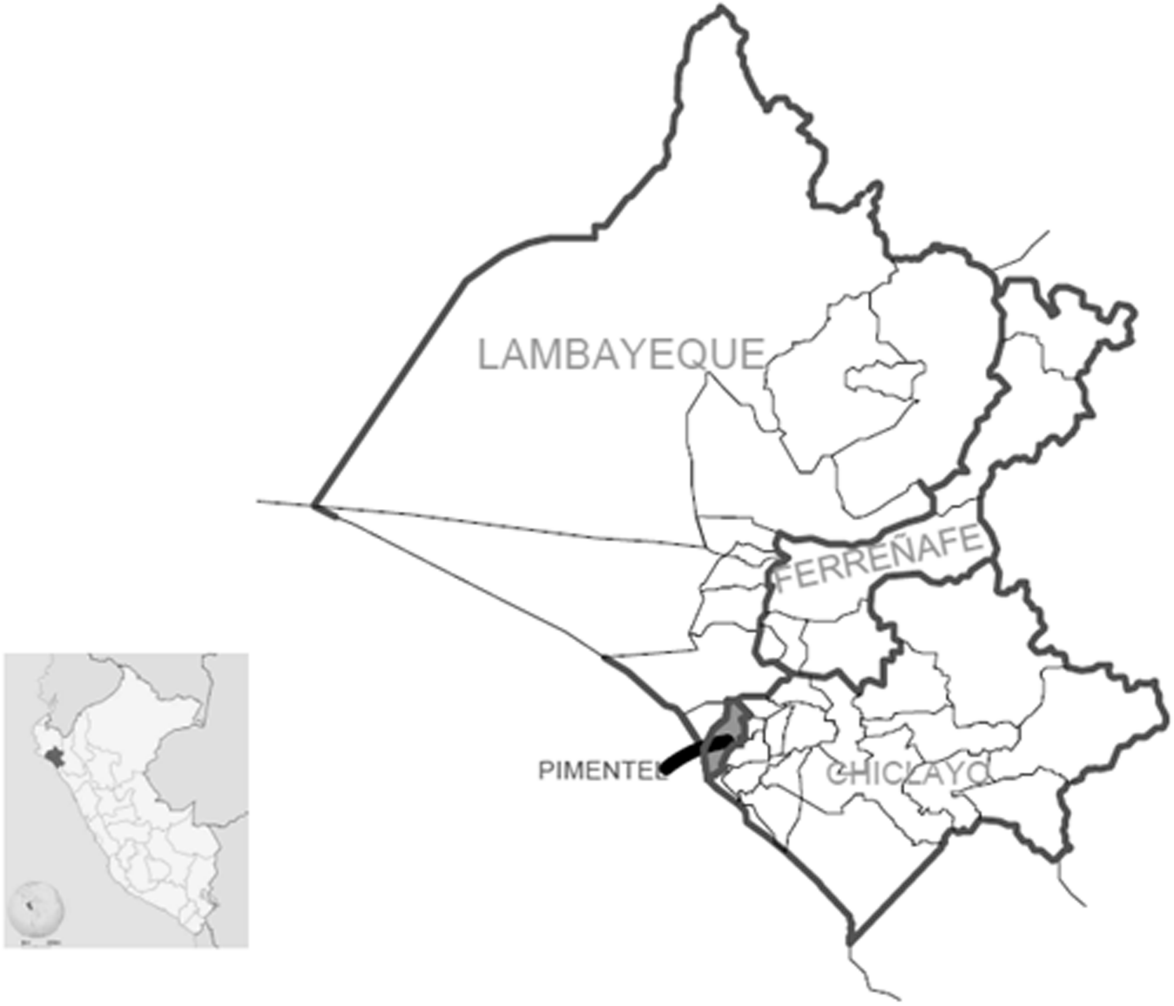
Map of the study area.

Pimentel is one of the main beaches in the northern Peru and belongs to Lambayeque, which is considered a semi-warm and tropical-dry region, with temperatures that exceed 30 °C during summer (Servicio Nacional de Meteorología e Hidrología, 2017; Oficina Nacional de Gobierno Electrónico e Informática, 2017) (Fig. 1). We surveyed all the beachgoers from 8 a.m. to 4 p.m. and from March 5 to March 19 (Peruvian summer, 2018) (Servicio Nacional de Meteorología e Hidrología, 2017). No sample was calculated. We surveyed all Spanish-speaking adults aged 18–59 who were in the study place within the specified time range.

### Variables and data collection

We applied the “Beach Questionnaire”, validated by De Troya-Martín et al. (2009) in a sample of Spanish beachgoers. This instrument aims to evaluate subjects’ behavior, attitudes and knowledge regarding sun exposure, and has been used in previous studies with similar populations (De Troya-Martín et al., 2014; De Troya-Martín et al., 2018). It has also been shown to be valid, reliable (Cronbach *α* > 0.7) and with good sensitivity to change (De Troya-Martín et al., 2009; Fernández-Morano et al., 2015).

The questionnaire included all our study variables and had the following sections: (1) Sociodemographic and academic data: sex, age, marital status, country of birth and educational level; (2) Color of non-sun-exposed skin: very fair, fair olive and dark; (3) Phototype: according to the Fitzpatrick model: I–IV, according to the erythema and tanning response after the first 60-min sun exposure in summer (De Troya-Martín et al., 2018); (4) Sun exposure habits on the beach in the last two summers: number of days spent at the beach each last two summers, number of hours per day and number of hours at midday (defined as between 12.00 and 16.00); (5) Sunburn history in the last summer (sunburn was defined as painful reddening of the skin) (De Troya-Martín et al., 2018); (6) Participants’ general knowledge about sun exposure with dichotomous response (true or false); (7) Attitudes related with sun exposure and sun protection, on a Likert-like scale of five categories (from “totally disagree” to “totally agree”) and (8) Sun protection practices.

### Statistical analysis

Collected data was entered into Microsoft Excel® with a double entry method to avoid errors during the process. After quality control, the database was exported to Stata version 13.0 (StataCorp LP, College Station, TX, USA).

We used relative and absolute frequencies to describe categorical variables and medians with interquartile ranges (after checking the absence of normality with Shapiro Wilk) for numerical variables. For bivariate analysis, we compared the categorical variables according to sex using the chi2 test. We considered a *P*-value < 0.05 as statistically significant.

### Ethics

This study was approved by the Institutional Review Board of the Hospital Nacional Docente Madre-Niño San Bartolome (RCEI-40), Lima, Peru. The participation was voluntary, and participants provided their informed oral consent, prior filling the survey. The anonymity of the participants and data confidentiality were ensured.

## Results

### Baseline characteristics of the study population

We surveyed a total of 410 beachgoers. The most frequent skin colors were olive (46.6%) and pale (35.4%). The most frequent Fitzpatrick phototype was type III (40.5%). Detailed sociodemographic and academic data are shown in Table 1.

**Table 1:** Sociodemographic, skin color and phototype data (*n* = 410).

**Characteristics**	***n*** (%)
Sex	
Male	176 (42.9)
Female	234 (57.1)
Age (years)^a^	28 (18–65)
Marital status	
Single	226 (55.1)
Married or living w/partner	175 (42.7)
Separated/Divorced	6 (1.5)
Widowed	3 (0.7)
Country of birth	
Peru	401 (98.1)
Argentina	2 (0.5)
Colombia	3 (0.7)
Ecuador	2 (0.5)
Mexico	1 (0.2)
Education	
None	4 (1.0)
Primary	11 (2.7)
Secondary	137 (33.4)
Higher Education	258 (62.9)
Skin color	
Very fair	22 (5.4)
Fair	145 (35.4)
Olive	191 (46.6)
Dark	52 (12.7)
Phototype	
I	62 (15.1)
II	79 (19.3)
III	166 (40.5)
IV	103 (25.1)

**Notes.**

a Median (Interquartile range).

### Sun-exposure habits and sunburns history

Men went to the beach more frequently in the last two summers (20.46% went more than 15 days vs 12.82% of women, *p* = 0.028). Likewise, 62.2% of the participants reported having suffered at least one sunburn last summer (Table 2).

**Table 2:** Sun-exposure habits and sunburns history.

**Item**	**Men**	**Women**	**Total**	***p***^a^
	***n*** (%)	***n*** (%)	***n*** (%)	
*In relation with the last two summers, choose…*				
Days of sun on the beach				
None	16 (9.09)	40 (17.09)	56 (13.66)	**0.028**
1–5	95 (53.98)	120 (51.28)	215 (52.44)	
6–15	29 (16.48)	44 (18.80)	73 (17.80)	
16–30	17 (9.66)	9 (3.85)	26 (6.34)	
>30	19 (10.80)	21 (8.97)	40 (9.76)	
Hours of sun exposure on the beach				
<30 min	26 (14.77)	41 (17.52)	67 (16.34)	0.706
30–1 h	43 (24.43)	52 (22.22)	95 (23.17)	
1–3 h	65 (36.93)	93 (79.74)	158 (38.54)	
>3 h	42 (23.86)	48 (20.51)	90 (21.95)	
Hours of sun at midday				
No sun	24 (13.64)	42 (17.95)	66 (16.10)	0.184
<1 h	48 (27.27)	42 (17.95)	90 (21.95)	
1–2 h	38 (21.59)	61 (26.07)	99 (24.15)	
2–4 h	39 (22.16)	56 (23.93)	95 (23.17)	
4–6 h	27 (15.34)	33 (14.10)	60 (14.63)	
*Last summer…*				
Sunburns				
None	63 (35.80)	92 (39.32)	155 (37.80)	0.818
1–2	73 (41.48)	100 (42.74)	173 (42.20)	
3–5	28 (19.51)	29 (12.39)	57 (13.90)	
6–10	5 (2.84)	5 (2.14)	10 (2.44)	
>10	7 (3.98)	8 (3.42)	15 (3.66)	

**Notes.**

a Chi2 test.

### Knowledge about sun exposure

Only the 13.66% of respondents (*n* = 56) correctly answered the seven questions related to sun exposure and skin cancer (Table 3). Individual analysis showed that the following questions had the lower percentage of correct answers: “Sun protection creams prevent aging of the skin produced by solar radiation” (60.0%) and “If I use total sun block I can sunbathe without any risk” (58.29%). Likewise, according to sex, significant differences were found in the response to “Once my skin is tanned, I don’t need to use sun protection cream” (76.14% of men answered correctly, versus 88.46% of women, *p* = 0.001).

**Table 3:** Participants’ general knowledge about sun exposure.

**Item**	**Men**	**Women**	**Total**	***p***^a^
	***n*** (%)	***n*** (%)	***n*** (%)	
Sun protection creams prevent aging of the skin produced by solar radiation				
True	107 (60.80)	139 (59.40)	246 (60.0)	0.776
False	69 (39.20)	95 (40.60)	164 (40.0)	
Sun is the main cause of skin cancer				0.452
True	163 (92.61)	221 (94.44)	384 (93.66)	
False	13 (7.39)	13 (5.56)	26 (6.34)	
Sun produces marks on the skin				0.135
True	152 (86.36)	213 (91.03)	365 (89.02)	
False	24 (13.64)	21 (8.97)	45 (10.98)	
If I use sunscreen I can sunbathe without any risk				
True	79 (44.89)	92 (39.32)	171 (41.71)	0.258
False	97 (55.11)	142 (60.68)	239 (58.29)	
Avoiding the midday sun (11–17 h) is the most efficient way of protecting my skin				
True	137 (77.84)	176 (75.21)	313 (76.34)	0.536
False	39 (22.16)	58 (24.79)	97 (23.66)	
Once my skin is tanned, I don’t need to use sun protection cream				
True	42 (23.86)	27 (11.54)	69 (16.83)	**0.001**
False	134 (76.14)	207 (88.46)	341 (83.17)	

**Notes.**

a Chi2 test.

### Attitudes related with sun exposure

More than three quarters of the respondents agreed or totally agreed that it is necessary to use sunscreen creams to avoid problems in the future (90.49%) and that its use is worthwhile despite not getting a tan (77.80%) (Table 4). Men were more frequently agreed that when they are tanned their clothes looks nicer (*p* = 0.048). Likewise, regarding the question “Sunbathing is relaxing”, men agreed or totally agreed with more frequency than women (63.64% vs. 46.15%, *p* < 0.001). The same thing happened with the item “Sunbathing improves my mood” (61.36% of men vs 49.15% of women, *p* = 0.014).

**Table 4:** Attitudes related with sun exposure.

**Item**	**Men**	**Women**	**Total**	***p***^a^
	***n*** (%)	***n*** (%)	***n*** (%)	
When I am tanned my clothes look nicer				
Totally agree/Agree	61 (34.66)	60 (25.64)	121 (29.51)	**0.048**
Indifferent/Disagree/Totally disagree	115 (65.34)	174 (74.36)	289 (70.49)	
Sunbathing helps prevent health problems				
Totally agree/Agree	76 (43.18)	109 (46.58)	185 (45.12)	0.494
Indifferent/Disagree/Totally disagree	100 (56.82)	125 (53.42)	225 (54.88)	
I like the feeling of the sun on my skin when I am lying on the beach				
Totally agree/Agree	64 (36.36)	65 (27.78)	129 (31.46)	0.064
Indifferent/Disagree/Totally disagree	112 (63.64)	169 (72.22)	281 (54.88)	
It is worth using sun protection cream to avoid future problems				
Totally agree/Agree	161 (91.48)	210 (89.74)	371 (90.49)	0.554
Indifferent/Disagree/Totally disagree	15 (8.52)	24 (10.26)	39 (9.51)	
I find sun protection creams unpleasant				
Totally agree/Agree	52 (29.55)	69 (29.49)	121 (29.51)	0.990
Indifferent/Disagree/Totally disagree	124 (70.45)	165 (70.51)	289 (70.49)	
It is worth using sun protection cream even though I don’t get a tan				
Totally agree/Agree	136 (77.27)	183 (78.21)	319 (77.80)	0.822
Indifferent/Disagree/Totally disagree	40 (22.73)	51 (21.79)	91 (22.20)	
People with a tan are more attractive				
Totally agree/Agree	67 (38.07)	79 (33.76)	146 (35.61)	0.367
Indifferent/Disagree/Totally disagree	109 (61.93)	155 (66.24)	264 (64.39)	
Sunbathing is healthy for my body				
Totally agree/Agree	95 (53.98)	107 (45.73)	202 (49.27)	0.098
Indifferent/Disagree/Totally disagree	81 (46.02)	127 (54.27)	208 (50.73)	
Sunbathing is relaxing				
Totally agree/Agree	112 (63.64)	108 (46.15)	220 (53.66)	<**0.001**
Indifferent/Disagree/Totally disagree	64 (36.36)	126 (53.85)	190 (46.34)	
Having a tan makes you look young and relaxed				
Totally agree/Agree	63 (35.80)	67 (28.63)	130 (31.71)	0.123
Indifferent/Disagree/Totally disagree	113 (64.20)	167 (71.37)	280 (68.29)	
Sunbathing improves my mood				
Totally agree/Agree	108 (61.36)	115 (49.15)	223 (54.39)	**0.014**
Indifferent/Disagree/Totally disagree	68 (38.64)	119 (50.85)	187 (45.61)	
I like sunbathing				
Totally agree/Agree	108 (61.36)	121 (51.71)	229 (55.85)	0.051
Indifferent/Disagree/Totally disagree	68 (38.64)	113 (48.29)	181 (44.15)	
When I go to the beach I prefer to be in the shade				
Totally agree/Agree	128 (72.73)	173 (73.93)	301 (73.41)	0.785
Indifferent/Disagree/Totally disagree	48 (27.27)	61 (26.07)	109 (26.59)	
I don’t like high-protection creams because they are anti-aesthetic				
Totally agree/Agree	53 (30.11)	62 (26.50)	115 (28.05)	0.420
Indifferent/Disagree/Totally disagree	123 (69.89)	172 (73.50)	295 (71.95)	

**Notes.**

a Chi2 test.

### Sun protection practices

The 63.9% of the respondents indicated that they usually or always use sunscreen when they go to the beach (Table 5). However, the compliance percentage was lower for the rest of the practices. Analysis by sex showed that women more frequently used sunshade (*p* = 0.001) and sunscreen (SPF ≥ 15) (*p* < 0.001).

**Table 5:** Sun protection practices.

**Item**	**Men**	**Women**	**Total**	***p***^a^
	***n*** (%)	***n*** (%)	***n*** (%)	
*When you go to the beach, you…*				
Use sunshade				
Always	37 (21.02)	92 (39.32)	129 (31.46)	**0.001**
Usually	27 (15.34)	27 (11.54)	54 (13.17)	
Sometimes	50 (28.41)	63 (26.92)	113 (27.56)	
Almost never	25 (14.20)	25 (10.68)	50 (12.20)	
Never	37 (21.02)	27 (11.54)	64 (15.61)	
Use sunglasses				
Always	35 (19.89)	65 (27.78)	100 (24.39)	0.406
Usually	23 (13.07)	28 (11.97)	51 (12.44)	
Sometimes	43 (24.43)	58 (24.79)	101 (24.63)	
Almost never	25 (14.20)	28 (11.97)	53 (12.93)	
Never	50 (28.41)	55 (23.50)	105 (25.61)	
Use hat or cap				
Always	57 (32.39)	73 (31.20)	130 (31.71)	0.718
Usually	28 (15.91)	35 (14.96)	63 (15.37)	
Sometimes	37 (21.02)	60 (25.64)	97 (23.66)	
Almost never	20 (11.36)	30 (12.82)	50 (12.20)	
Never	34 (19.32)	36 (15.38)	70 (17.07)	
Wear long sleeves or long trousers				
Always	22 (12.50)	27 (11.54)	49 (11.95)	0.742
Usually	17 (9.66)	16 (6.84)	33 (8.05)	
Sometimes	29 (16.48)	48 (20.51)	77 (18.78)	
Almost never	33 (18.75)	45 (19.23)	78 (19.02)	
Never	75 (42.61)	98 (41.88)	173 (42.20)	
Avoid sun 12.00–16.00				
Always	38 (21.59)	60 (25.64)	98 (23.90)	0.802
Usually	32 (18.18)	44 (18.80)	76 (18.54)	
Sometimes	61 (34.66)	72 (30.77)	133 (32.44)	
Almost never	15 (8.52)	23 (9.83)	38 (9.27)	
Never	30 (17.05)	35 (14.96)	65 (15.85)	
Use sunscreen (SPF ≥ 15)				
Always	54 (30.68)	125 (53.42)	179 (43.66)	<**0.001**
Usually	37 (21.02)	46 (19.66)	83 (20.24)	
Sometimes	42 (23.86)	43 (18.38)	85 (20.73)	
Almost never	14 (7.95)	10 (4.27)	24 (5.85)	
Never	29 (16.48)	10 (4.27)	39 (9.51)	

**Notes.**

SPF Sun Protection Factor

a Chi2 test.

## Discussion

### Sun-exposure habits and sunburns history

In our study, we found that men went with more frequency to the beach than women, which may be related to recreational activities that are often performed at the place of study (e.g., surfing and soccer). This finding differs from that found by Fernández-Morano et al. (2014). In their study, women went to the beach more frequently (75.5% compared to 66.4% of men) (Fernández-Morano et al., 2014). However, this may be because its population was comprised only of adolescents, which may be related to another of their findings, which was a higher likelihood for sunbathing and tanning by the female group.

We found that more than 60% had suffered at least one sunburn in the last summer, a percentage higher than those reported in studies conducted in the US (Buller et al., 2011; Holman et al., 2014) and Europe (De Troya-Martín et al., 2018; Haluza et al., 2016; Kritsotakis et al., 2016). This may be due to the lack of education in the local population, which negatively affects their practices and habits regarding sun exposure. This finding is a call for the implementation of intervention and education strategies, since it has been demonstrated that a personal sunburns history is strongly associated with skin cancer (Erdmann et al., 2013; Garbe & Leiter, 2009; Sánchez, Nova & De la Hoz, 2012; Wu et al., 2016).

### Participants’ general knowledge about sun exposure

Less than 15% of the respondents correctly answered the seven questions about sun exposure. This lack of knowledge could be a possible explanation for the growing trend of skin cancer in the Peruvian population (Sordo & Gutiérrez, 2013). Studies conducted in adolescents and adults beachgoers in Spain have reported better levels of knowledge (Fernández-Morano et al., 2014; Cercato et al., 2015), which could be a reflection of the positive impact of the interventions and campaigns that have been carried out in that country (De Troya-Martín et al., 2014; Fernández-Morano et al., 2015; Del Boz et al., 2015).

Some studies suggest that a good level of knowledge about sun exposure may not always go hand in hand with adequate attitudes or practices  (Haluza, Simic & Moshammer, 2016; Mousavi et al., 2011; Yan et al., 2015). However, a systematic review showed that some sun protection behaviors were positively associated with a good level of knowledge about skin cancer (Day et al., 2014). Also, a study conducted by De Troya-Martín et al. (2018) reported an important role of knowledge about sun exposure in the prevention of sunburns.

### Attitudes related with sun exposure

Most of the participants presented good attitudes regarding the use of sunscreen. These results are more favorable to those found by Mousavi et al. (2011) and Fernández-Morano et al. (2017). A possible explanation may be that during the last summer, high temperature peaks were reported in comparison to previous years, as well as heavy rains on the northern coast of Peru (Servicio Nacional de Meteorología e Hidrología, 2017). To face the problem, intervention, reconstruction and prevention activities were carried out, including information campaigns which were disseminated by local and national media.

On the other hand, men presented inappropriate attitudes more frequently, which differs from the results reported in two studies conducted in Spain (Fernández-Morano et al., 2017; Fernández-Morano et al., 2014). A possible explanation may lie in the continuing influence of social media on current stereotypes and the perception of beauty and body image concerns (Fardouly & Vartanian, 2016; Barlett, Vowels & Saucier, 2008). In this sense, since tanning has usually been related to concepts of beauty, our result could be understood a little more by the fact that nowadays men are increasingly adopting some attitudes that were previously prioritized by women, such as sunbathing and tanning (Fernández-Morano et al., 2017; Haluza et al., 2016).

### Sun protection practices

More than half of the participants reported a frequent sunscreen use (usually or always). This result is similar to that found by Devos et al. (2012) in a sample of beachgoers from the northern coast of Belgium, and better than those reported in other studies conducted in Europe (Fernández-Morano et al., 2014; Weinstock et al., 2000) and Asia (Mousavi et al., 2011; Yan et al., 2015). However, the percentages of compliance for the other practices were less than 50%. Since current literature mentions that sunscreen use alone is not enough to control the skin exposure to UVR (Diffey, 2001; Iannacone, Hughes & Green, 2014; Grossman et al., 2018), beachgoers should adopt other measures, such as avoiding midday, wearing hat/cap and long-sleeved clothes, seeking for shade and skin self-examination (Molho-Pessach & Lotem, 2007; Grossman et al., 2018; Skotarczak et al., 2015; Mancebo, Hu & Wang, 2014).

Women had more and better sun protection practices, mainly related to the use of sunscreen and sunshade. Previous research have also reported better sun exposure behaviors and practices in this population (Weinstock et al., 2000; Yan et al., 2015; Devos et al., 2012; Olsen et al., 2015). This may be linked to the attitudes that, according to our study, were also better in women. In addition, this could explain why sunburns are more frequent in men, according to some studies (De Troya-Martín et al., 2018; Reuter et al., 2010; Kasparian, McLoone & Meiser, 2009).

### Relevance and implications

Skin cancer has become a major public health problem (Erdmann et al., 2013; Garbe & Leiter, 2009; Guy et al., 2015). In recent years, its global incidence rates have increased (Glazer et al., 2017; Arnold et al., 2014; Schmerling et al., 2011), and Peru is not the exception (Sordo & Gutiérrez, 2013). Thus, awareness of the risks of sun exposure and adequate sun-protective behaviors and attitudes are needed. Our results, however, are not as favorable as expected.

Evidence suggest that Public Health efforts should encourage sun-safety precautions to avoid UVR overexposure (Haluza et al., 2016; Blumthaler, 2018). In addition, the beach seems to be an ideal setting for promoting adequate sun-protective behaviors (Cercato et al., 2015). In this sense, prevention, detection and intervention campaigns related to sun protection and skin cancer should be carried out, as they have shown satisfactory results in other studies (Pagoto, McChargue & Fuqua, 2003; De Troya-Martín et al., 2014; Del Boz et al., 2015; Emmons et al., 2011).

### Limitations

Some limitations must be highlighted. First, we used self-report questions; despite using a validated instrument to measure our variables, social desirability bias might arise. Second, we did not address some variables that could potentially influence the results of our study, such as current or previous illnesses and family history of skin cancer. Finally, the extrapolation of our results is limited to the Pimentel beachgoers. However, given that it is the busiest beach in Lambayeque, it gives us a good approximation to the possible reality in the region.

## Conclusion

Only one or two out of ten respondents correctly answered all the questions related to sun exposure knowledge. Negative attitudes were more frequent in men, and women presented better practices. Future research should study other variables that are also related to sun protection. Thus, interventions could be more targeted and with even more promising results. Finally, we recommend that future studies develop and evaluate the impact of sun-protective interventions, as previous research have shown their potential to promote sun protection in recreational settings (Hay et al., 2017; Rodrigues et al., 2017; Rodrigues, Sniehotta & Araujo-Soares, 2013).

## Additional Information and Declarations

### Data Availability

The following information was supplied regarding data availability: Toro-Huamanchumo, Carlos (2018): Dataset.eng - Sun exposure. figshare. Dataset. https://doi.org/10.6084/m9.figshare.6828725.v1.
